# Lymph Node Yield and Disease-Free Survival After Neoadjuvant Chemoimmunotherapy for Esophageal Squamous Cell Carcinoma: A Multicenter, Retrospective Cohort Study

**DOI:** 10.1245/s10434-026-19753-4

**Published:** 2026-07-01

**Authors:** Huilai Lv, Zuopeng Wang, Xuefeng Leng, Jinbo Zhao, Changchun Wang, Jiangbo Lin, Xinyu Mei, Jianfei Shen, Yingnan Yang, Ping Zhang, Xiaolong Yan, Zhenhua Li, Mingbo Wang, Chunyue Gai, Yongtao Han, Zhigang Li, Lianmei Zhao, Xufeng Guo, Ziqiang Tian

**Affiliations:** 1 Department of Thoracic Surgery, The Fourth Hospital of Hebei Medical University, Shijiazhuang, Hebei Province China; 2 Hebei Key Laboratory of Accurate Diagnosis and Comprehensive Treatment of Esophageal Cancer, Shijiazhuang, Hebei Province China; 3 Department of Thoracic Surgery, Sichuan Clinical Research Center for Cancer, Chengdu, Sichuan Province China; 4 Department of Thoracic Surgery, Tangdu Hospital, Air Force Medical University, Xian, Shanxi Province China; 5 Department of Thoracic Surgery, Zhejiang Cancer Hospital, Hangzhou, Zhejiang Province China; 6 Department of Thoracic Surgery, Fujian Medical University Union Hospital, Fuzhou, Fujian Province China; 7 Department of Thoracic Surgery, The First Affiliated Hospital of University of Science and Technology of China, Hefei, Anhui Province China; 8 Department of Thoracic Surgery, Taizhou Hospital of Zhejiang Province Affiliated to Wenzhou Medical University, Linhai, Zhejiang Province China; 9 Department of Thoracic Surgery, Harbin Medical University Cancer Hospital, Harbin, Heilongjiang Province China; 10 Department of Radiotherapy, The Fourth Hospital of Hebei Medical University, Shijiazhuang, Hebei Province China; 11 Department of Thoracic Surgery, Shanghai Chest Hospital, Shanghai Jiao Tong University School of Medicine, Shanghai, China; 12 Research Center, The Fourth Hospital of Hebei Medical University, Shijiazhuang, Hebei Province China

**Keywords:** Lymph node yield, Esophageal squamous cell carcinoma, Neoadjuvant chemoimmunotherapy, Esophagectomy, Disease-free survival

## Abstract

**Background:**

The optimal extent of lymphadenectomy following neoadjuvant chemoimmunotherapy (nCIT) for esophageal squamous cell carcinoma (ESCC) remains unclear. Current recommendations are largely derived from neoadjuvant chemoradiotherapy cohorts, and their applicability in the era of immunotherapy is unclear. This study evaluated the association between lymph node dissection (LND) yield and disease-free survival (DFS) in patients with ESCC treated with nCIT.

**Methods:**

This retrospective multicenter cohort study included 465 patients with ESCC who underwent nCIT followed by radical esophagectomy at six hospitals in China between January 2019 and December 2023. The median follow-up was 40.7 months. The total number of dissected lymph nodes was analyzed in relation to DFS. The restricted mean survival time at 5 years was estimated by using random survival Forest-based models, with subgroup analyses by posttherapy pathological stage (ypT/ypN).

**Results:**

A higher lymph node yield was associated with improved DFS, although the relationship was nonlinear and varied by pathological subgroup. In patients with ypT0–2N0 disease, DFS improved with increasing LND up to approximately 20–30 lymph nodes, after which the benefit plateaued. In patients with residual nodal disease (ypN1–3), higher lymph node yields were associated with longer DFS, with greater yields observed in more advanced disease stages. Across all subgroups, lower lymph node yields were consistently associated with inferior DFS.

**Conclusions:**

In this multicenter cohort of patients with ESCC treated with nCIT, lymph node yield was associated with DFS in a stage-dependent manner, suggesting its potential role as a postoperative quality indicator for surgical lymphadenectomy after nCIT.

**Supplementary Information:**

The online version contains supplementary material available at https://doi.org/10.1245/s10434-026-19753-4.

In recent decades, the treatment paradigm for esophageal squamous cell carcinoma (ESCC) has evolved from surgery alone to multimodal strategies that incorporate neoadjuvant chemotherapy (nCT) or neoadjuvant chemoradiotherapy (nCRT) followed by esophagectomy. Lymph node dissection (LND) remains a key component of surgical management and has long been associated with staging accuracy and oncological outcomes in patients undergoing primary surgery for ESCC.^1^ However, the appropriate extent of lymphadenectomy after neoadjuvant therapy remains an area of ongoing debate.

The current clinical practice guidelines reflect this uncertainty. The National Comprehensive Cancer Network (NCCN) recommends extensive lymphadenectomy during radical esophagectomy, with a minimum retrieval of 15 lymph nodes (LNs), although these recommendations are largely derived from cohorts treated with nCRT rather than immunotherapy-based regimens.^2^ Evidence from secondary analyses, including the NEOCRTEC5010 trial, suggests that retrieval of ≥ 20 LNs may be associated with improved survival in patients with ESCC treated with nCRT.^3^ However, whether these benchmarks are applicable in the context of neoadjuvant chemoimmunotherapy (nCIT) is unknown.

The introduction of programmed cell death protein-1 (PD-1) inhibitors has substantially altered the neoadjuvant treatment landscape of ESCC. Emerging studies suggest the potential efficacy of nCIT, as well as its superior safety profile and advantages in reducing nodal disease burden.^4,5^ These immunotherapy-related effects may influence both lymph node status and interpretation of lymph node yield, raising questions about the transferability of lymphadenectomy targets established in the pre-immunotherapy era.

Given the absence of evidence-based guidance on lymph node yield following nCIT, the present multicenter study evaluated the association between lymph node yield and disease-free survival (DFS) in patients with ESCC treated with nCIT followed by esophagectomy. This study explored postoperative lymph node yield thresholds that may serve as benchmarks for surgical lymphadenectomy in this evolving treatment setting.

## Methods

### Study Design and Patient Population

This multicenter retrospective cohort study included consecutive patients with ESCC who received nCIT followed by radical esophagectomy at six tertiary medical centers in China between January 2019 and December 2023. The study was conducted in accordance with the Declaration of Helsinki and approved by the Ethics Committee of the Fourth Hospital of Hebei Medical University (Approval No. 2025KS063). The requirement for informed consent was waived because of the retrospective nature of the study.

### Race and Ethnicity Data

Race and ethnicity data were not collected in this study. All patients were treated at tertiary medical centers in China, and the study population was ethnically homogeneous, consisting predominantly of individuals of Han Chinese ethnicity. Therefore, race/ethnicity was not analyzed as a study variable.

### Inclusion and Exclusion Criteria

Patients were eligible for inclusion if they met the following criteria: (1) primary tumor located within the thoracic esophagus; (2) receipt of neoadjuvant chemoimmunotherapy followed by radical esophagectomy with histopathological confirmation of ESCC; (3) achievement of R0 resection; (4) surgical approach limited to McKeown, Sweet, or Ivor-Lewis procedures; (5) availability of complete demographic, clinical, pathological, treatment, and follow-up data; and (6) absence of distant metastases as determined by preoperative imaging and postoperative pathological evaluation.

Patients were excluded if they had any of the following: (1) a prior history of or concurrent diagnosis of another malignancy; (2) incomplete pathological data precluding adequate assessment; (3) perioperative mortality, defined as death within 30 days after surgery or during the same hospitalization; or (4) loss to follow-up.

### Neoadjuvant Chemoimmunotherapy and Surgical Procedures

All patients received nCIT according to institutional protocols. nCIT consisted of chemotherapy combined with immune checkpoint inhibitors, administered according to institutional protocols. Surgical resection was performed after completion of neoadjuvant therapy, with the choice of surgical approach (McKeown, Sweet, or Ivor-Lewis) determined by tumor location, surgeon judgment, and institutional practice.

Standard esophagectomy with systematic lymphadenectomy was performed in all the patients. The extent of lymph node dissection (LND) was not protocol-mandated and reflected real-world surgical practice. Lymph node yield was defined as the total number of lymph nodes retrieved and was examined pathologically from surgical specimens.

### Pathological Evaluation and Follow-up

Resected specimens were examined by pathologists at each participating center as part of routine clinical care. For study purposes, all patients were uniformly reviewed and restaged by two experienced thoracic surgeons and pathologists according to the eighth edition of the American Joint Committee on Cancer (AJCC)/Union for International Cancer Control (UICC) staging system, with postneoadjuvant pathological staging recorded as ypT and ypN categories. Pathological complete response (pCR) was defined as the absence of residual viable tumor cells in both the primary tumor site and resected lymph nodes (ypT0N0).

Patients were followed up at regular intervals after surgery according to institutional protocols. Disease-free survival was defined as the time from the date of surgery to the first documented recurrence or death from any cause, whichever occurred first.

### Statistical Analysis

Patients were stratified into six groups based on LN yield: 1–9, 10–19, 20–29, 30–39, 40–49, and ≥ 50 LNs. Baseline characteristics were summarized by using descriptive statistics for the LN yield groups. Continuous variables are reported as medians with interquartile ranges (IQRs), and categorical variables are presented as frequencies and percentages. Statistical analyses were performed by using R software (version 4.4.3; R Foundation for Statistical Computing, Vienna, Austria), and data management was performed by using the *dplyr* package.^6^

Given prior evidence suggesting that the association between LN yield and survival may be nonlinear, a machine learning–based analytical framework was applied to model DFS across varying extents of lymphadenectomy. First, a quantile random forest approach (*quantregForest* package^7^) was used to define patient-specific plausible ranges of LN yield based on demographic, clinical, and pathological variables. Second, a random survival Forest-based virtual twin method was applied to estimate the DFS across the individualized LN yield ranges. The restricted mean survival time (RMST) at 5 years was used as the primary summary measure to quantify the survival differences associated with different LN yields. Survival curves were visualized using the *ggplot2* package.^8^ The Lowess method was applied solely for graphical presentation to facilitate visual interpretation and did not influence any statistical inferences.^9^

Analyses were performed for the overall cohort and stratified by pathological ypT stage, ypN stage, and combined ypT–ypN subgroups. Detailed descriptions of the machine learning methodology, model tuning, and validation procedures are provided in the supplementary information. Where applicable, statistical significance was evaluated using two-sided tests with a *p*-value of < 0.05. All analyses were exploratory and hypothesis-generating rather than supporting causal inference.

## Results

### Patient Characteristics

**Fig. 1:**
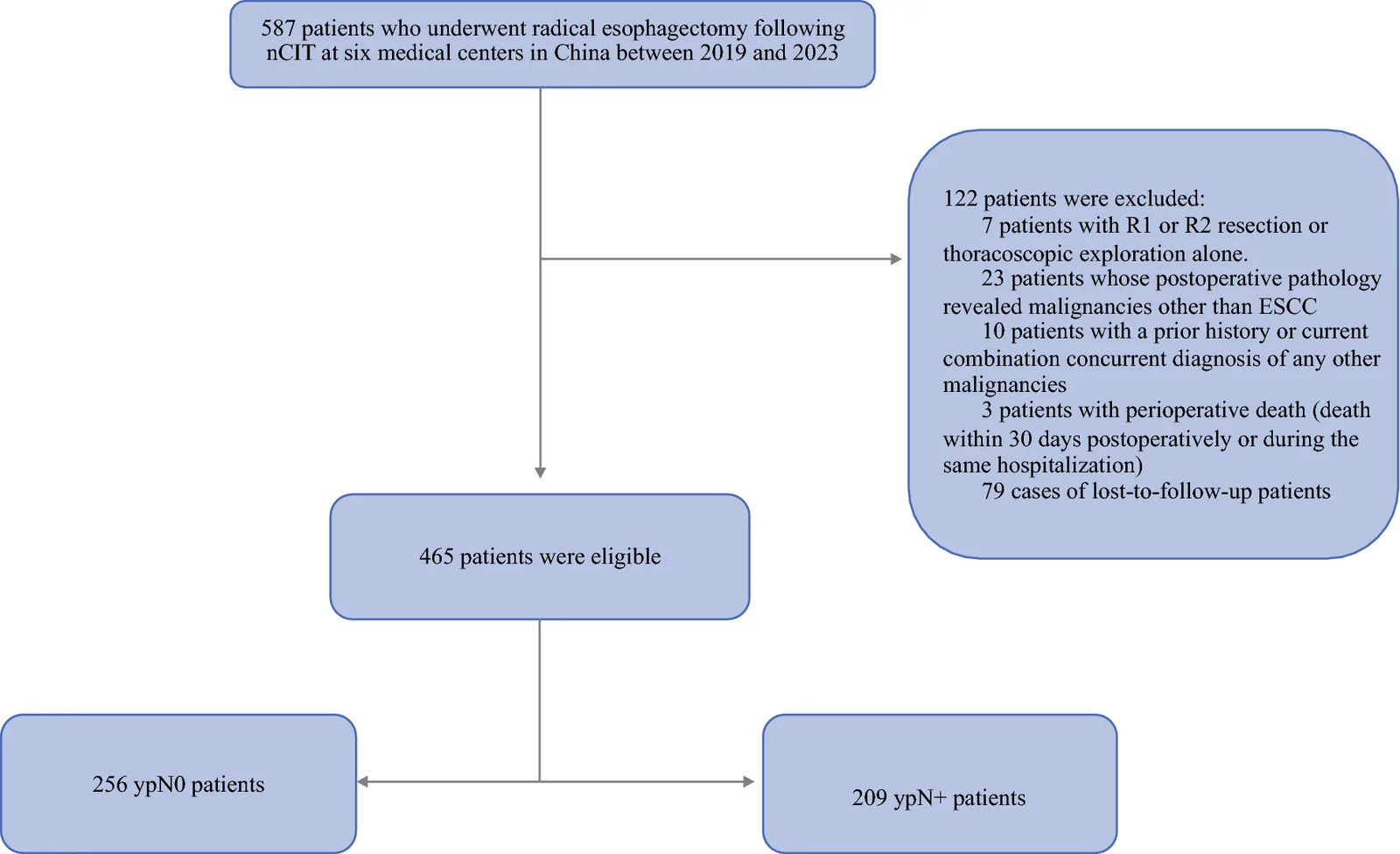
Patient selection for the multicenter retrospective cohort study

A total of 587 patients were screened, and 465 eligible patients with ESCC who received nCIT followed by esophagectomy were included in the final analysis (Fig. 1). The median follow-up duration was 40.7 months (interquartile range [IQR] 36.4–47.8). Of these patients, 256 (55.1%) were classified as ypN0 and 209 (44.9%) as ypN+ after neoadjuvant therapy. The baseline clinicopathological characteristics are summarized in Table 1 and Supplemental Table S1, and the postoperative complications are presented separately in Table 2.

**Table 1:** Baseline clinicopathological characteristics of patients with esophageal squamous cell carcinoma

Characteristics	Overall (n = 465)	ypN0 (n = 256)	ypN+ (n = 209)
Age, yr	62.45 ± 7.08	62.78 ± 7.3	62.04 ± 6.81
Male	371 (79.8)	197 (77.0)	174 (83.3)
Female	94 (20.2)	59 (23.0)	35 (16.7)
BMI, kg/m^2^	23.03 ± 3.05	22.88 ± 3.09	23.21 ± 2.99
Smoking: No	214 (46.0)	119 (46.5)	95 (45.5)
Smoking: Yes	251 (54.0)	137 (53.5)	114 (54.5)
Alcohol: No	241 (51.8)	142 (55.5)	99 (47.4)
Alcohol: Yes	224 (48.2)	114 (44.5)	110 (52.6)
ASA score 0	352 (75.7)	195 (76.2)	157 (75.1)
ASA score 1	94 (20.2)	52 (20.3)	42 (20.1)
ASA score 2	17 (3.7)	8 (3.1)	9 (4.3)
ASA score 3	1 (0.2)	0 (0.0)	1 (0.5)
ASA score 4	1 (0.2)	1 (0.4)	0 (0)
McKeown	387 (83.2)	209 (81.6)	178 (85.2)
Ivor–Lewis	43 (9.2)	25 (9.8)	18 (8.6)
Sweet	35 (7.5)	22 (8.6)	13 (6.2)

*BMI* body mass index, *ASA* American Society of Anesthesiologists

**Table 2:** Postoperative complications

Complication	Overall (n = 465)	ypN0 (n = 256)	ypN+ (n = 209)
Pneumonia	129 (27.7)	65 (25.4)	64 (30.6)
Pneumothorax	11 (2.4)	5 (2.0)	6 (2.9)
Respiratory failure	42 (9.0)	26 (10.2)	16 (7.7)
Massive pleural effusion	142 (30.5)	77 (30.1)	65 (31.1)

### Extent of Lymph Node Dissection

The number of dissected lymph nodes (LNs) varied substantially across individuals (range 2–92; median yield of 24 LNs). Variations in lymph node yield were observed across surgical approaches with median LN counts of 26 for the McKeown procedure, 20 for the Sweet procedure, and 17 for the Ivor-Lewis procedure. The proportion of patients with ≥ 30 dissected LNs ranged from 6 to 40% across all procedures (Supplemental Table S2), indicating heterogeneity in lymphadenectomy practice.

### Lymphadenectomy in the Overall Population

**Fig. 2:**
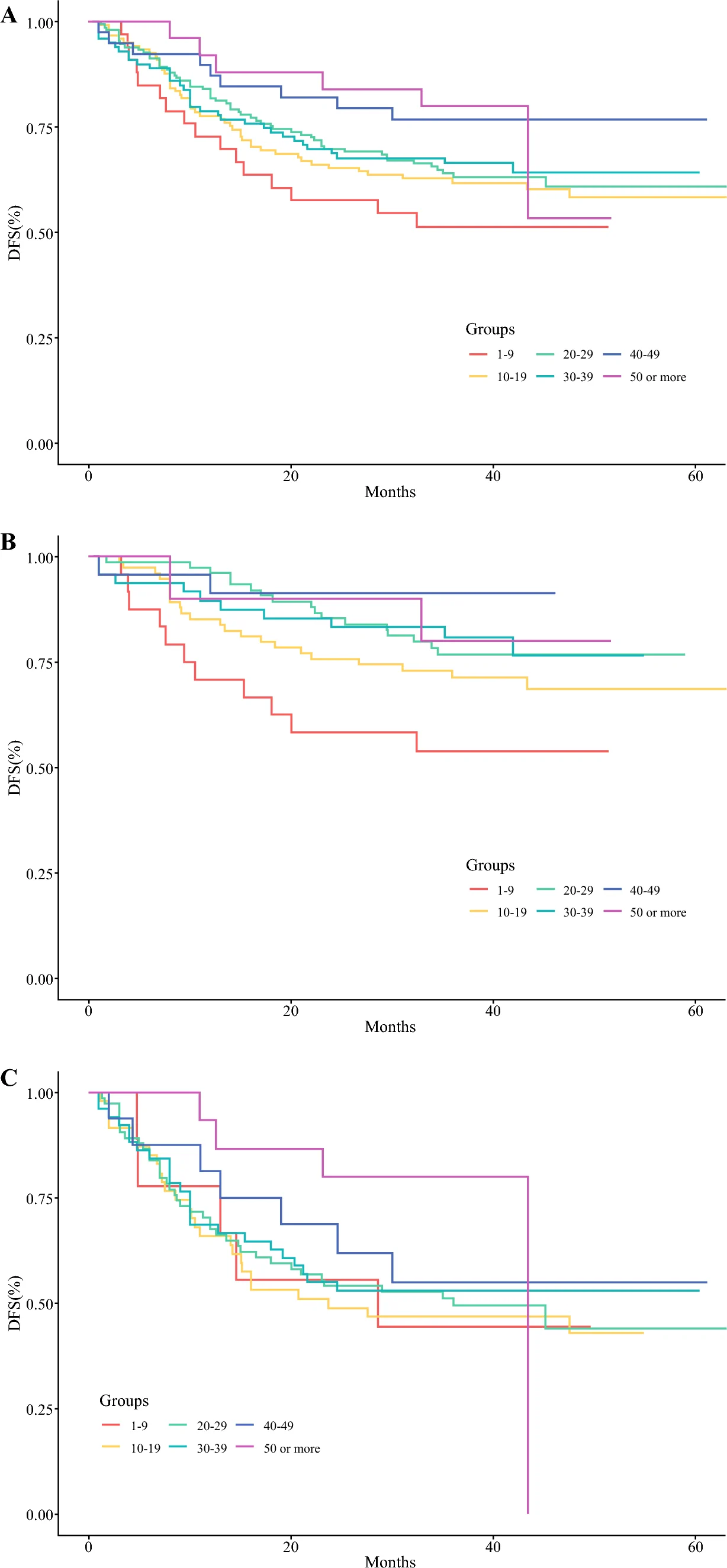
Kaplan-Meier curves for disease-free survival (DFS) stratified by lymph node yield categories (1–9, 10–19, 20–29, 30–39, 40–49, and ≥50 dissected lymph nodes) in (**A**) the overall cohort, (**B**) patients with ypN0 disease, and (**C**) patients with ypN+ disease

Patients were stratified into six groups according to LN yield (1–9, 10–19, 20–29, 30–39, 40–49, and ≥ 50 LNs). Unadjusted Kaplan–Meier analysis demonstrated an overall association between higher LN yield and improved DFS in the overall patients, ypN0 patients, and ypN+ patients (Fig. 2).

**Fig. 3:**
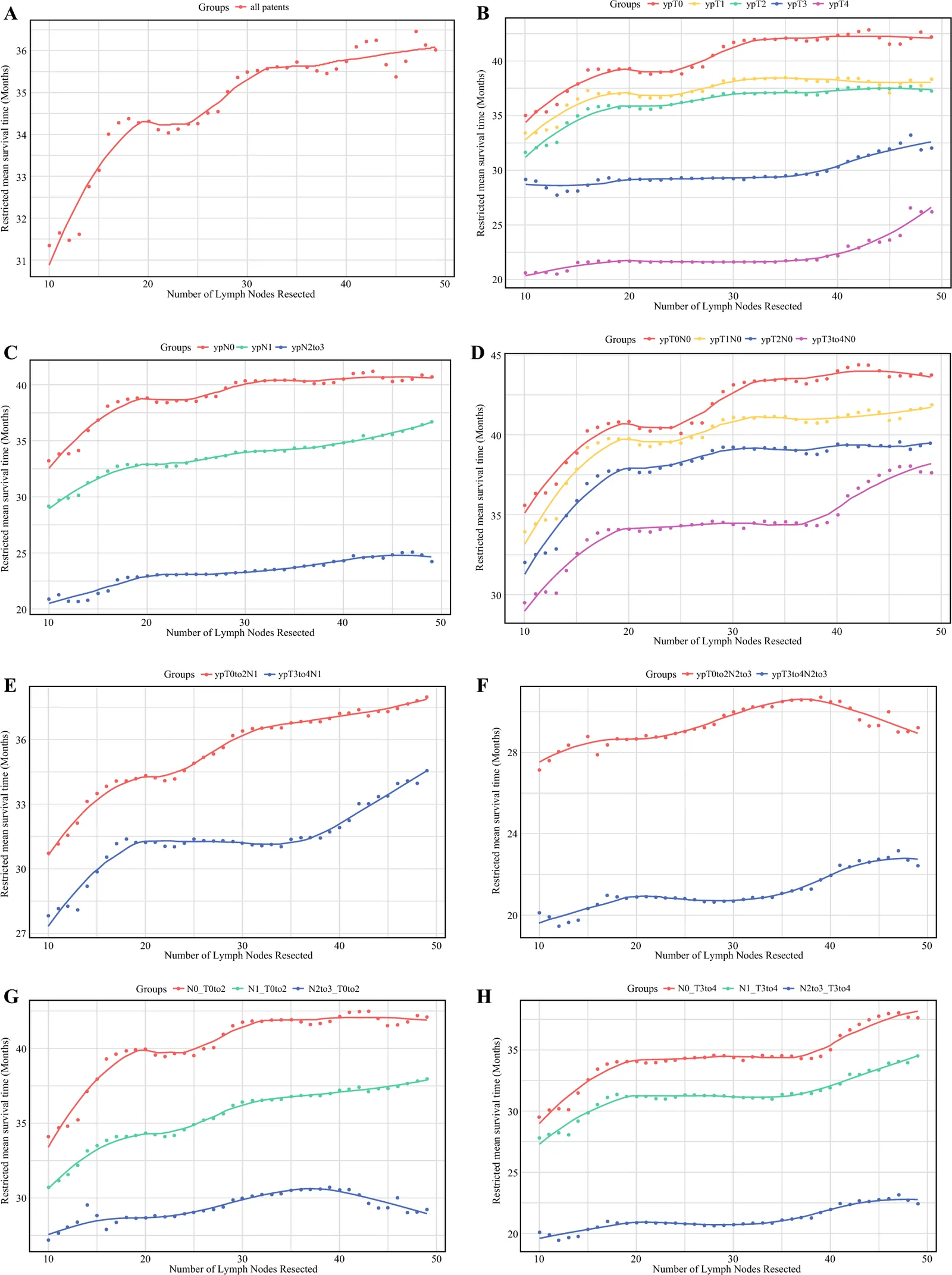
Restricted mean survival time (RMST) at 5 years according to lymph node yield, estimated using random survival forest–based models. (**A**) Overall study population. (**B**) Stratified by postoperative ypT stage. (**C**) Stratified by postoperative ypN stage. (**D–H**) Stratified by the combined ypT–ypN subgroups

The RMST analysis revealed a nonlinear association between LN yield and DFS in the overall cohort (Fig. 3A). The RMST increased most markedly as the LN yield increased from 10 to 19, followed by a slower increase in the 20–29 range. Beyond approximately 30 dissected LNs, the RMST curve exhibited a plateau-like pattern, with only modest additional gains observed with further increases in the LN yield. Given this overall nonlinear pattern, we further explored whether the association between LN yield and survival differed across the pathological subgroups defined by ypT and ypN stages.

### Association Between Lymph Node Yield and DFS by ypT and ypN Stage

Across pathological subgroups defined by ypT and ypN stages, a higher LN yield was generally associated with a longer RMST (Fig. 3B, C). Among patients with ypT0–T2 disease, the RMST increased with LN yield up to approximately 20 dissected lymph nodes, followed by a more gradual increase at higher yields. In contrast, among patients with ypT3–T4 disease, the RMST continued to increase across a wider range of LN yields, with more pronounced gains observed beyond 40 dissected lymph nodes (Fig. 3B).

When stratified by nodal status (Fig. 3C), patients with ypN0 disease demonstrated increasing RMST with higher LN yields up to approximately 20 lymph nodes, after which the survival benefit appeared to attenuate. In patients with residual nodal disease (ypN1–3), the RMST increased across a broader range of LN yields without a clearly defined plateau.

### Lymph Node Yield Across Combined ypT–ypN Subgroups

Further stratification by the combined ypT–ypN stage revealed substantial heterogeneity in the association between LN yield and RMST (Fig. 3D–H). In patients with ypT1–2N0 disease, the RMST increased with LN yield up to approximately 20 LNs, with minimal additional benefit observed at higher yields (Fig. 3D).

Among patients who achieved a pathological complete response (pCR), the RMST increased with LN yield up to approximately 30 LNs and subsequently plateaued (Fig. 3D). In contrast, in patients with ypT3–4N0 disease, higher LN yields were associated with longer RMST, with continued improvement observed at yields exceeding 40 LNs (Fig. 3D, H).

In patients with residual nodal disease (ypN+), the RMST generally increased with LN yield across most subgroups (Fig. 3E–H). Notably, in ypT0–2N2–3 patients, RMST reached its highest values within the 30–40 LN range (Fig. 3F, G), whereas in ypT3–4N2–3 patients, the RMST continued to increase with higher LN yields (Fig. 3F, H), indicating stage-dependent patterns in the association between LN yield and the RMST.

**Fig. 4:**
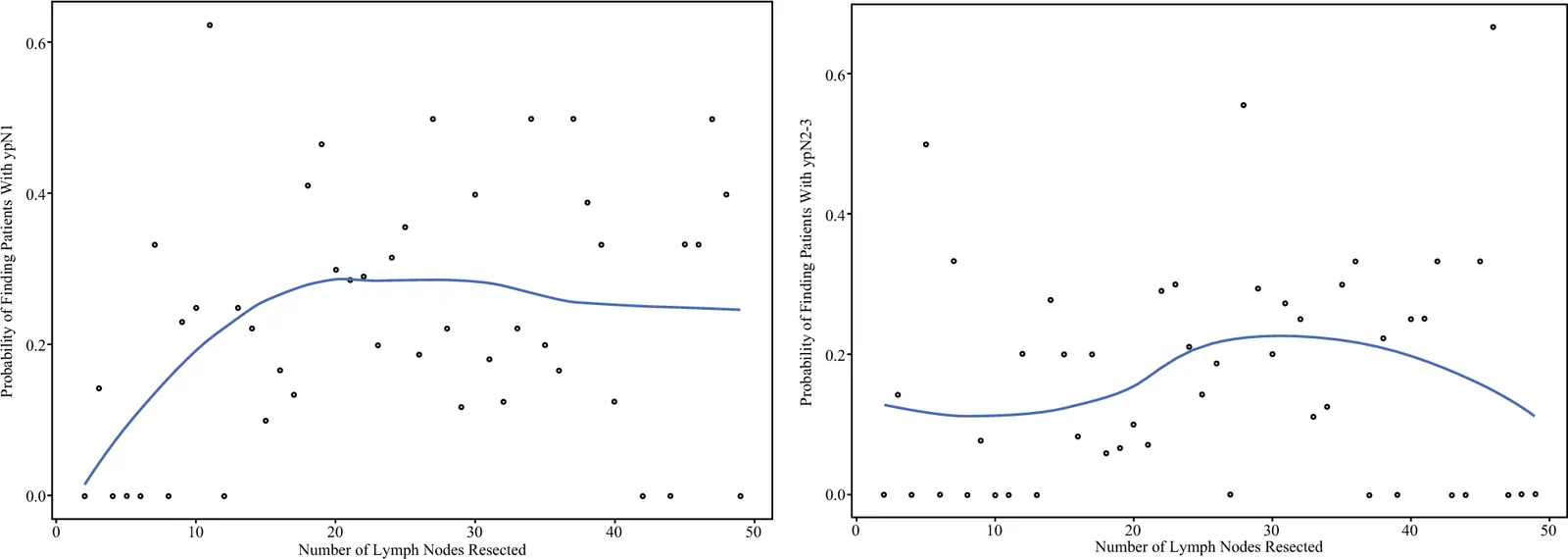
Probability of identifying pathologically positive lymph nodes as a function of lymph node yield, stratified by postoperative nodal status

Figure 4 illustrates the proportion of patients with pathologically positive lymph nodes across increasing LN yields, providing complementary information on nodal assessment rather than survival outcomes. The probability of identifying nodal metastases increased with LN yield up to an intermediate range, particularly among patients with ypN1 and ypN2–3 disease and plateaued or declined at higher LN yields. These patterns suggest that adequate, but not unlimited, lymphadenectomy may improve nodal assessment in patients with residual nodal involvement.

## Discussion

This multicenter retrospective study evaluated the association between lymph node yield and DFS in patients with ESCC treated with nCIT followed by esophagectomy. The results demonstrated that lymph node yield is associated with DFS in a nonlinear and stage-dependent manner, with substantial heterogeneity across pathological subgroups defined by ypT and ypN status.

Rather than indicating a uniform survival benefit from increasingly extensive lymphadenectomy, our findings suggest that the magnitude and pattern of the association between lymph node yield and DFS differ according to tumor depth and nodal status. In the overall cohort, survival gains associated with higher lymph node yields appeared to attenuate beyond intermediate ranges, consistent with prior observations in neoadjuvant-treated ESCC populations. These results highlight the complexity of interpreting lymph node yields in postneoadjuvant immunotherapy settings.

Lymph node yield has been widely regarded as a surrogate marker for multiple unmeasured factors, including surgical technique, pathological processing, and institutional quality of care.^10,11^ In the context of nCIT, immunotherapy-induced nodal sterilization and regression may further influence both nodal burden and the interpretation of lymph node counts. Previous studies have shown that the distribution and detectability of lymph node metastases differ substantially after neoadjuvant treatment, complicating the direct extrapolation of lymphadenectomy benchmarks derived from surgery-alone or chemoradiotherapy-based cohorts.^12,13^

Previous studies have consistently reported associations between a higher lymph node yield and improved survival in esophageal cancer, although most have modeled this relationship as linear.^14^ Our analysis extends these observations by demonstrating nonlinear phase-dependent associations between lymph node yield and DFS across pathological subgroups. Such patterns may partly explain why prior studies reported heterogeneous lymph node thresholds and inconsistent conclusions regarding the “optimal” extent of lymphadenectomy.

Stratified analyses indicated that lymph node yield behaved differently across ypT and ypN categories, consistent with earlier reports showing stage-specific variations in lymphadenectomy–survival associations.^15,16^ In patients without residual nodal disease (ypN0), survival gains were primarily observed at low-to-intermediate lymph node yields, after which the benefit plateaued. In contrast, patients with residual nodal involvement demonstrated survival gains across a broader range of lymph node yields. These findings align with the hypothesis that lymph node yield reflects both staging adequacy and potential therapeutic clearance of residual micrometastatic disease, particularly in patients with limited nodal burden, although the relative contributions of these mechanisms cannot be disentangled in an observational study.^1,17^

Importantly, our results should not be interpreted as prescriptive recommendations for intraoperative decision-making. Pathological ypT and ypN status is determined postoperatively, and lymph node yield is influenced by multiple factors that cannot be standardized during surgery. Accordingly, the lymph node yield ranges identified in this study are best interpreted as postoperative benchmarks reflecting surgical and pathological thoroughness rather than targets to be actively pursued during the esophagectomy.

nCRT and nCIT differ substantially in their mechanisms of action and effects on lymph node pathology. nCRT primarily induces tumor cell death through radiation-mediated DNA damage and cytotoxic chemotherapy, often resulting in fibrosis and structural alterations of lymphatic tissues. In contrast, nCIT exerts its effects through immune checkpoint inhibition, enhancing antitumor immune responses, and promoting immune-mediated tumor clearance, which may alter the distribution and detectability of lymph node metastases.

These differences have potential implications for lymphadenectomy strategies. In the context of nCIT, preservation of the regional immune architecture may be relevant for postoperative immune surveillance and responsiveness to subsequent therapies. Therefore, lymph node dissection after nCIT may require a balance between oncologic clearance and preservation of immune function. This may partly explain the stage-dependent variation in lymph node yield thresholds observed in our study and highlights the need for cautious extrapolation of lymphadenectomy benchmarks derived from nCRT-treated populations.^18–21^

Comparisons with prior studies on nCRT–treated populations further highlight the heterogeneity of lymphadenectomy effects across treatment contexts. Although some similarities exist, differences in mechanisms of action, tumor biology, treatment response, and metastatic patterns limit the generalizability of lymph node yield thresholds across regimens and histologies.^22–25^ Therefore, extrapolation of specific benchmarks should be approached with caution.

This study has several limitations. First, lymph node counting practices may vary across institutions and pathologists, contributing to the measurement variability in the lymph node yield. Second, the sample size limited the precision of certain ypT–ypN subgroup analyses, and sparse data on extreme lymph node yields may have affected the stability of the modeled survival estimates. Third, as a retrospective observational study, residual confounding cannot be excluded, and the observed associations should not be interpreted as causal. Finally, the machine learning–based analytical approach was exploratory in nature and not externally validated. Confirmation in independent cohorts is therefore warranted.

## Conclusions

In this multicenter cohort of patients with esophageal squamous cell carcinoma treated with neoadjuvant chemoimmunotherapy followed by esophagectomy, lymph node yield was associated with disease-free survival (DFS) in a complex, nonlinear, and stage-dependent manner. These findings suggest that lymph node yield may serve as a postoperative benchmark reflecting the thoroughness of surgical lymphadenectomy and pathological assessment, rather than as a prescriptive intraoperative target. Further prospective studies are warranted to validate these observations and define clinically meaningful quality indicators in the immunotherapy era.

## Supplementary Information

Below is the link to the electronic supplementary material.

Supplementary file1 (DOCX 136 kb)

## Data Availability

The datasets used and/or analyzed in the current study are available from the corresponding author upon reasonable request.
