# Supplementary material for: Lymph Node Yield and Disease-Free Survival After Neoadjuvant Chemoimmunotherapy for Esophageal Squamous Cell Carcinoma: A Multicenter, Retrospective Cohort Study
Source: Ann Surg Oncol. 2026 Jul 1;33(10):8864–73. doi: 10.1245/s10434-026-19753-4 (PMC13585866; doi:10.1245/s10434-026-19753-4)
Supplement: Supplementary file 1 [file 10434_2026_19753_MOESM1_ESM.docx]

**Supplemental Material**

| **Supplementary Appendixes** |  |
| --- | --- |
| **Appendix 1.** Analytical strategy | *page. 2* |
| **Supplementary Tables and Figures** |  |
| **Table S1.** Clinical and post-neoadjuvant therapy pathological (yp) cancer characteristics | *page. 8* |
| **Table S2.** Information on the number of lymph nodes dissected by centers and surgical approaches | *page. 10* |
| **Fig. S1.** Example of how range of plausible extent of lymphadenectomy was determined | *page. 11* |
| **Fig. S2.** Extent of lymphadenectomy | *page. 12* |

**Supplementary Appendixes**

**Appendix 1. *Analytical strategy***

An increase in the extent of lymph node dissection (LND) has been shown to be associated with a progressive increase in survival. However, the effect appears to be non-linear.^1^ To explore the complex relationship between LND and prognosis, we therefore introduced a new statistical method in our analysis, which was accomplished by the following steps:

1) Determining the reasonable extent of LND.

Quantile random forest regression was used to determine the plausible range of LND based on patient demographics (including age [y], sex, body mass index [kg/m^2^], smoking history, drinking history, and surgical approach) and tumor pathological characteristics (including cT, cN, ypT, ypN, number of positive lymph nodes, histologic grade, tumor location, number of dissected lymph nodes, and invasive characteristics [LVI，PNI]).^2, 3^

The detailed methodology is provided as follows, and an illustrative example is presented in Fig. S1. In this study, we utilized quantile random forest regression as a methodology for the estimation of the association between patient, clinical and cancer variables and the number of regional lymph nodes (LNs) resected.^2^ This approach was then employed to predict the probability of each whole number of LNs to be resected. The analysis was conducted utilizing the quantile random forest function in the randomForestSRC R package.^4^ The objective of quantile random forest is to estimate conditional distribution function of the number of nodes resected. Y is defined as the number of resected nodes. A quantile random forest can be defined as a collection of random regression trees such that, for a given patient covariate X = x, it provides an estimate for the conditional distribution function of Y given X = x.

$$\text{FY}│\text{X (y) = P [Y }\leq\text{ y}│\text{X = x]}$$

The conditional density is defined as

$$\begin{matrix} \text{f Y}│\text{X (y) = P [Y = y}│\text{X = x]} \\ \text{ = P [Y }\leq\text{ y}│\text{X = x] - P [Y }\leq\text{ y-1 }│\text{X = x]} \\ \text{ = FY}│\text{X (y) - FY}│\text{X (y - 1)} \end{matrix}$$

Therefore, upon being provided with FY│X, the density of Y│X can invariably be ascertained. This methodology is employed by quantile random forest.

Note that quantile regression, which estimates the conditional distribution function, differs from usual regression, where the goal is to estimate the conditional expectation of Y. Random forests regression^3^ used 1,000 bootstrap regression trees split using mean-squared-error. Node size of a tree was set at 5 observations; random feature selection was set to 5 (5 of the 15 variables were randomly selected at each tree node for splitting).

In order to ascertain the range of LN resection that is plausible for a patient, we define that the lower limit of the range is the first instance in which the patient’s density for number of resected nodes exceeds the QL-quantile for population. In our research, QL was set to 0.025. The upper limit was determined by identifying the last instance when the patient’s cumulative distribution function was no larger than a preset value of Qu, which was set to Qu = 0.975. This defined the region of plausible number of lymph node dissections, as illustrated by typical cumulative distribution functions in Supplemental Fig. S1, which shows the range of plausible extent of lymphadenectomy.

Because machine learning algorithms, which consider the influence of algorithms, account for outliers and model interpretability, we qualified the LND range based on the available data. The minimum plausible number of resected LNs was set to 1 and the maximum was truncated at 60 (Supplemental Fig. S2). This predicted interval is referred to as the “plausible LND range”.

2) Individualized survival analysis was performed for each patient.

For each patient, we conducted survival analyses using an extended method of random survival forest (RSF), incorporating interactions between all variables and the actual number of LNs removed.^5, 6^ A novel causal inferential approach based on an extension of RSF, random survival forest-virtual twin with interaction (RSF-VT-I),^5^ was applied to nonparametrically model the the different factor levels of the relevant pathological characteristic variables, such as cT, cN, ypT and ypN, were converted into dummy variables. These dummy variables were then combined with the number of LNDs as interaction terms. This approach engendered a substantial degree of flexibility, thereby facilitating enhanced estimation of the survival function in the context of potentially intricate interactions between the extent of lymphadenectomy with cancer characteristics. The resulting data set contained 41 variables. The RSF was fit using the dataset, with disease-free survival (DFS) as the outcome. computations were implemented using randomForestSRC R software.^4^ To this end, 1,000 bootstrap survival trees were grown under log-rank splitting, employing a tree node size of 10, with 15 features selected at random for candidates to split each tree node. This is larger than the default setting, equal to the square root of the number of features; a larger value was used due to the large number of features. Utilizing the forest constructed, RSF-VT-I yielded an out-of-sample estimate. yielded an out-of-sample estimate of the survival function for each patient. However, each patient possesses only the single actual number of LNs resected, yet individual treatment effect (ITE) estimation necessitates predicted values of survival for all plausible numbers of nodes resected. Consequently, for each patient, a sequence of survival curves is predicted by replacing the counterfactual numbers with the possible LND range of the same patient (predicted by quantile random forest regression). Under the assumption of weak unconfoundedness, RSF-VT-I-estimated survival curves yield unbiased estimators of the ITE for a patient. However, as with the use of propensity scores to provide unbiased estimates of the average treatment effect (ATE), it is imperative to circumscribe the potential range of LND to a reasonable extent.

RSF-virtual twin (RSF-VT) approach was used to predict the survival corresponding to the actual number of LNs removed, based on the RSF model. The virtual twin method was then employed to predict the survival curves for each individual across the “plausible LND range”, generating a sequence of up to 60 survival curves. Each curve in the sequence represents an individual’s projected survival for a potential number of dissected LNs.

3) Determining the optimal extent of LND.

A critical aspect of our study was the selection of appropriate metrics to quantify the patient survival benefits. Historically, many researchers have assumed a linear relationship between the extent of LND and survival, employing methods such as Kaplan-Meier curves, Cox regression models, and other analytical techniques. Metrics such as hazard ratios (HRs), and median survival times were used to assess potential survival benefits. However, these metrics are susceptible to violations of the proportional hazard assumption. Therefore, we calculated the restricted mean survival time (RMST), defined as the area under the survival curve from the time of surgery to a specific postoperative time point.^7-9^ This resulted in a set of RMST values for each patient’s survival curve sequence, with each RMST value reflecting the projected survival time at a given LND number. RMST values were then grouped by the number of dissected LNs, with mean RMST calculated per group to generate scatterplots illustrating the relationship between the extent of LND and survival. The 5-year postoperative period was selected for this analysis. The optimal LND range was defined as the range that yielded the highest survival benefit.

**REFERENCES**

1. Guo X, Wang Z, Yang H, ET al. Impact of lymph node dissection on survival after neoadjuvant chemoradiotherapy for locally advanced esophageal squamous cell carcinoma: from the results of neocrtec5010, a randomized multicenter study. *Ann Surg.* 2023;277:259-266. <http://doi.org/10.1097/sla.0000000000004798>

2. Meinshausen N. Quantile regression forests. *J Mach Learn Res.* 2006;7:983-999.

3. Breiman L. Random forests. *Machine Learning.* 2001;45:5-32. <http://doi.org/10.1023/A:1010933404324>

4. Ishwaran H, Kogalur UB. randomForestSRC: random forests for survival, regression and classification (RF-SRC). R package version 2.5.0. <http://cran.r-project.org>. Published 2017.

5. Lu M, Sadiq S, Feaster DJ, Ishwaran H. Estimating individual treatment effect in observational data using random forest methods. *J Comput Graph Stat.* 2018;27:209-219. <http://doi.org/10.1080/10618600.2017.1356325>

6. Published online October 31, 2012 Randomforestsrc: fast unified random forests for survival, regression, and classification (rf-src). Available.

7. Royston P, Parmar MK. The use of restricted mean survival time to estimate the treatment effect in randomized clinical trials when the proportional hazards assumption is in doubt. *Stat Med.* 2011;30:2409-2421. <http://doi.org/10.1002/sim.4274>

8. Andersen PK, Hansen MG, Klein JP. Regression analysis of restricted mean survival time based on pseudo-observations. *Lifetime Data Anal.* 2004;10:335-350. <http://doi.org/10.1007/s10985-004-4771-0>

9. Zhao L, Claggett B, Tian L, et al. On the restricted mean survival time curve in survival analysis. *Biometrics.* 2016;72:215-221. <http://doi.org/10.1111/biom.12384>

**Supplementary Tables and Figures**

**Table S1.** Clinical and post-neoadjuvant therapy pathological (yp) cancer characteristics

| **Characteristics** | **No. (%)** |
| --- | --- |
| **cT stage** |  |
| T1 | 9 (1.9) |
| T2 | 45 (9.7) |
| T3 | 351 (75.5) |
| T4 | 60 (12.9) |
| **cN stage** |  |
| N0 | 122 (26.2) |
| N1 | 211 (45.4) |
| N2 | 115 (24.7) |
| N3 | 17 (3.7) |
| **ypT stage** |  |
| T0 | 135 (29.0) |
| T1 | 97 (20.9) |
| T2 | 67 (14.4) |
| T3 | 145 (31.2) |
| T4 | 21 (4.5) |
| **ypN stage** |  |
| N0 | 256 (55.2) |
| N1 | 121 (26.0) |
| N2 | 73 (15.7) |
| N3 | 15 (3.2) |
| **PNI** |  |
| No | 403 (86.7) |
| Yes | 62 (13.3) |
| **LVI** |  |
| No | 377 (81.1) |
| Yes | 88 (18.9) |
| **Grade** |  |
| G0 | 141 (30.3) |
| G1 | 52 (11.2) |
| G2 | 161 (34.6) |
| G3 | 111 (23.9) |
| **Location** |  |
| Upper | 65 (14.0) |
| Middle | 209 (44.9) |
| Lower | 191 (41.1) |
| **pCR** |  |
| No | 366 (78.7) |
| Yes | 99 (21.3) |

**PNI**: Perineural Invasion; **LVI**: Lymph-Vascular Invasion; **pCR:** Pathologically Complete Response

**Table S2. Information on the number of lymph nodes dissected by centers and surgical approaches**

|  | **No. of patients** |  | **No. of patients with  >30 nodes resected** |  | **Number of  lymph nodes dissected** |
| --- | --- | --- | --- | --- | --- |
|  | **No. (%) or median (P25, P75)** | | | | |
| **Center** |  |  |  |  |  |
| 1 | 53 (11.4) |  | 10 (18.9) |  | 22 (15, 26) |
| 2 | 81 (17.4) |  | 12 (14.8) |  | 18 (11, 25) |
| 3 | 38 (8.2) |  | 14 (36.8) |  | 23 (17, 32) |
| 4 | 133 (28.6) |  | 63 (47.4) |  | 29 (22, 36) |
| 5 | 93 (20.0) |  | 9 (9.7) |  | 18 (14, 22) |
| 6 | 67 (14.4) |  | 54 (80.6) |  | 38 (31, 44) |
| **Surgical approach** |  |  |  |  |  |
| McKeown | 387 (83.2) |  | 155 (40.1) |  | 26 (18, 35) |
| Ivor-Lewis | 43 (9.2) |  | 3 (6.9) |  | 17 (12, 22) |
| Sweet | 35 (7.5) |  | 4 (11.4) |  | 20 (10, 25) |

**Fig. S1. Determination of the plausible range of lymph node dissection.**

A quantile random forest model was trained to predict the distribution of lymph node dissection counts for an individual patient based on demographic and pathological characteristics. Predicted quantiles were used to construct a conditional cumulative distribution function and corresponding density function of lymph node yield. The lower bound of the plausible lymphadenectomy range (QL) was defined as the minimum lymph node count corresponding to a cumulative probability ≥0.025, and the upper bound (QU) as the maximum lymph node count corresponding to a cumulative probability ≤0.975. These bounds defined the patient-specific plausible range of lymph node dissection used for subsequent analyses.

**Fig. S2. Distribution of lymph node yield.**

Each dot represents the number of patients with a given lymph node yield. The final category represents patients with ≥60 lymph nodes resected. Among the 465 patients, 33 (7.1%) had 1–9 nodes resected; 121 (26.0%) had 10–19 nodes resected; 149 (32.0%) had 20–29 nodes resected; 99 (21.2%) had 30–39 nodes resected; 39 (8.4%) had 40–49 nodes resected; 15 (3.2%) had 50–59 nodes resected; and 9 (2.1%) had ≥60 nodes resected.
